# EMT-Related Gene Signature Predicts the Prognosis in Uveal Melanoma Patients

**DOI:** 10.1155/2022/5436988

**Published:** 2022-08-12

**Authors:** Yufei Lv, Lixian He, Mengyi Jin, Wenxin Sun, Gang Tan, Zuguo Liu

**Affiliations:** ^1^Postdoctoral Station for Basic Medicine, Hengyang Medical School, University of South China, Hengyang, Hunan 421001, China; ^2^The First Affiliated Hospital, Department of Ophthalmology, Hengyang Medical School, University of South China, Hengyang, Hunan 421001, China; ^3^Department of Ophthalmology, Xiang'an Hospital of Xiamen University, Fujian Provincial Key Laboratory of Ophthalmology and Visual Science, Fujian Engineering and Research Center of Eye Regenerative Medicine, Eye Institute of Xiamen University, School of Medicine, Xiamen University, Xiamen, Fujian 361100, China; ^4^Xiamen University Affiliated Xiamen Eye Center, Xiamen, Fujian 361005, China

## Abstract

**Background:**

Uveal melanoma (UVM) is the most common primary intraocular malignancy in adults. Epithelial-mesenchymal transition (EMT) is an essential regulator of the UVM's immune microenvironment. However, the precise role of EMT in UVM remains to be explored and the development of a related treatment strategy is urgently needed.

**Methods:**

Multiomics data and clinical information for TCGA-UVM were used to identify the EMT subtypes and analyze their regulatory role in the immune microenvironment in UVM. A machine-learning method based on the identified subtypes was utilized to construct the EMT feature-based prognostic model. External validation cohorts GSE84976 and GSE22138 were employed to validate the model's robustness. Immunotherapy cohort IMvigor210 was used to explore the model's potential to predict immunotherapy responsiveness.

**Results:**

Two EMT subtypes were identified in UVM. The role of EMT in shaping the immune microenvironment and regulating cancer-immunity circle of UVM was analyzed. A robust prognostic model was presented and validated to predict patient prognosis. The model also predicted patient's immune features and immunotherapy responsiveness.

**Conclusion:**

The EMT-mediated immune features in UVM were illustrated, providing a reliable model to facilitate precise UVM treatment. This research may assist in decision-making during clinical UVM therapy.

## 1. Introduction

As the most common primary intraocular malignancy in adults, uveal melanoma (UVM) represents 85% of ocular melanomas [1, 2]. UVM arises from melanocytes of the uveal tract. About 85%–90% of UVM cases originate from the choroid, while the remaining occur in the iris or ciliary body [3]. Traditional first-line therapy strategy, including radiotherapy, surgical treatment, and enucleation, has reached a satisfactory rate of local disease control and long-term survival [4]. However, due to the high metastatic rate and high mortality rate secondary to metastasis, traditional therapy strategies for systemic UVM treatment remain abundant [5, 6]. Targeting therapy and immunotherapy in recent years have become the emerging components of systemic UVM treatment and have resulted in an impressive therapeutic effect in clinical practice [7–10]. However, a robust method to help identify the UVM patients and potentially obtain satisfactory clinical benefits is still lacking. The underlying mechanism of the therapy's nonresponse in UVM needs to be further explored.

Epithelial-mesenchymal transition (EMT) is a process in which epithelial cells lose their junctions and polarity and acquire the characteristics of migratory mesenchymal cells [11]. This phenomenon of cellular plasticity usually takes place during the embryo development. However, it can also be observed during cancer progression [11, 12]. A tumor can obtain a greater migration capability and is more likely to result in distant metastasis via EMT [13]. EMT can also contribute to the formation of immune-suppressive microenvironment and raise the activity of the immune checkpoints [14]. Furthermore, EMT is correlated with the activation of tumor drug efflux pumps and antiapoptotic effects [15]. In these mechanisms, an EMT-active tumor will have more aggressive clinicopathological features and a lower responsiveness to traditional antitumor drugs and can be a potential candidate for immunotherapy [16–19].

Therefore, a robust method to help estimate UVM EMT activity in order to develop a more appropriate multidisciplinary therapy strategy for patients is urgently needed. In this research study, genomic information for UVM samples derived from The Cancer Genome Atlas (TCGA) was employed to comprehensively assess the EMT activity and illustrate the tumoral microenvironment characteristics. Two UVM subtypes were identified: EMT inactive and EMT active. These two subtypes demonstrated a distinct pattern in clinicopathological features, somatic mutation features, immune microenvironment features, and prognosis. To further facilitate the clinical application, an EMT feature-based prognostic model to predict UVM prognosis and responsiveness for targeting therapy and immunotherapy was developed using the least absolute shrinkage and selection operator (LASSO) Cox regression. Transcriptome and somatic mutation data acquired from multiple external validation cohorts were employed to comprehensively evaluate the prognostic model's efficiency. The results demonstrated that the model has great potential to be utilized as a decision-making tool to assist doctors in evaluating tumor aggressiveness and choosing optimal therapy strategy during a precise UVM treatment.

## 2. Materials and Methods

### 2.1. Data Acquisition and Processing

TCGA-UVM patient clinical information, RNA-seq data, and gene mutation data were acquired from TCGA database (https://portal.gdc.cancer.gov) and processed using *R* packages “TCGAbiolinks” and “maftools” [20–22]. Transcriptome data and clinical follow-up information of GSE84976 and GSE22138 cohorts were acquired from the National Center for Biotechnology Information (NCBI) Gene Expression Omnibus (GEO) database (https://www.ncbi.nlm.nih.gov/gds). Immunotherapy cohort data were acquired from published literature and obtained by the *R* package “IMvigor210CoreBiologies” [23]. All clinical data utilized in this research are publicly available. Thus, local ethical approval was not required.

### 2.2. EMT Signature Analysis and Identification of the UVM Subtype

To identify the EMT inactive subtype and EMT active subtype in UVM and to conduct further analysis, 18 EMT signatures were acquired from three published sources [24–26]. EMT signature interaction was analyzed based on the Pathway Commons (https://www.pathwaycommons.org/) database and visualized using Cytoscape software. The EMT signature's coexpression status was analyzed using the Pearson correlation and visualized with *R* package “ggcorrplot”. The EMT signature's protein-protein interaction (PPI) network was constructed using Cytoscape software to visualize the landscape of the EMT signature-related protein interaction. *K*-means clustering based on these EMT signatures was used to identify the EMT inactive and active subtypes with *R* package “pheatmap.” To further validate the robustness of the subtype identification, EMT subtype activity was estimated using Gene Set Variation Analysis (GSVA) according to the EMT signatures [27].

### 2.3. Analysis of the Subtypes' Immune Characteristics

To quantify the samples' immune cell infiltration levels, CIBERSORT based on the CIBERSORT tool (https://cibersort.stanford.edu/) was utilized. Samples' immune score, stromal score, and ESTIMATE score were calculated in *R* package “ESTIMATE” to evaluate the samples' immune activity [28]. The process consisted of seven steps during anticancer immune response. The steps include the release of cancer cell antigens (Step 1), cancer antigen presentation (Step 2), priming and activation (Step 3), trafficking of immune cells to tumors (Step 4), infiltration of immune cells into tumors (Step 5), recognition of cancer cells by T cells (Step 6), and killing of cancer cells (Step 7). The cancer-immunitycircle-related pathway was acquired from the published literature. Detailed pathway information is presented in Table S1. The pathway activity was evaluated using GSVA in *R* package “GSVA” [27].

### 2.4. Acquisition and Analysis of DEGs between the EMT Inactive Subtype and the EMT Active Subtype

Subtypes of differentially expressed genes (DEGs) were acquired using the threshold of |logFC| > 1 and adj − *P* < 0.01 with *R* package “limma.” To investigate the DEG-mediated biological function, enrichment analysis was conducted based on the Kyoto Encyclopedia of Genes and Genomes (KEGG) and Gene Ontology (GO) database [29, 30]. The enriched terms were visualized, and clustering was analyzed using Metascape (https://metascape.org/).

### 2.5. Construction of EMT Feature-Based Prognostic Model

To construct the EMT feature-based prognostic model, patient outcome-related genes were first identified among all DEGs. Univariate Cox regression was employed to analyze the gene impact on patient outcomes. The result with *P* < 0.001 was selected. Next, the selected genes were analyzed using LASSO Cox regression to further screen the candidate genes for model construction and to calculate the coefficient of the selected genes. Finally, the EMT feature-based prognostic model (EMT score) was developed based on the selected genes and their coefficients. All analyses were conducted by the *R* package “glmnet.”

### 2.6. Statistical Analysis

The *K*–*M* survival analysis combined with a log-rank test was used to analyze the differences in patient prognosis between the two groups. TimeROC analysis was performed to evaluate the prediction accuracy of the EMT score. The Wilcoxon rank sum test was used to compare the continuous variables between the two groups. The differences in sample distribution in the two groups were analyzed with a chi-square test. If not specifically mentioned, *P* < 0.05 was considered statistically significant.

## 3. Results

### 3.1. Identification of the EMT Inactive Subtype and the EMT Active Subtype in UVM

**Figure 1:**
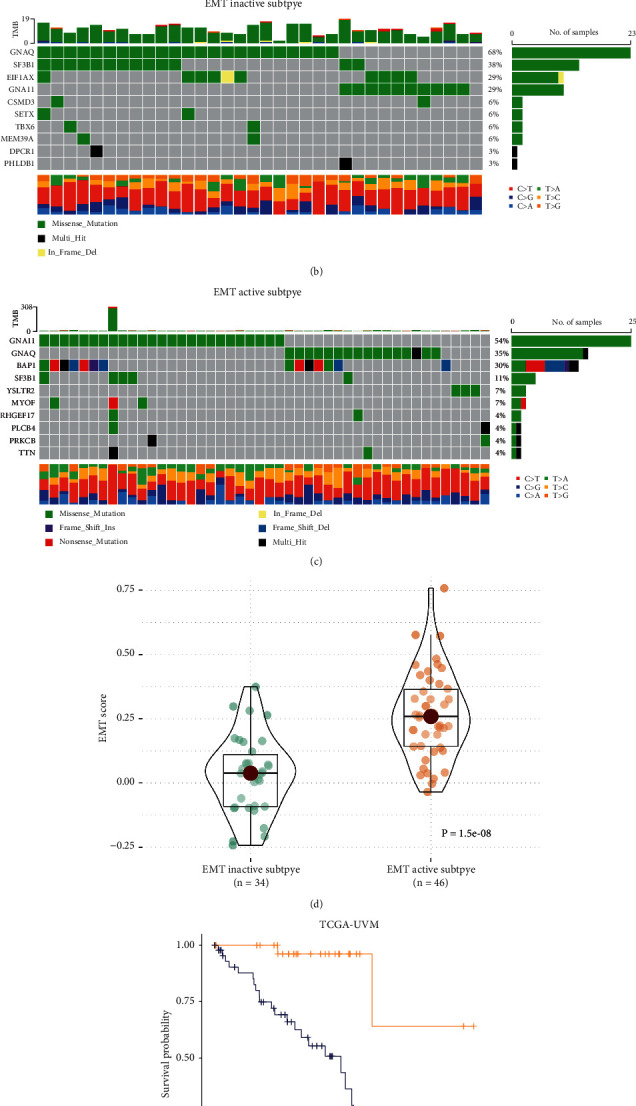
Identification of UVM's EMT inactive subtype and EMT active subtype. (a) Landscape of UVM EMT subtypes and clinicopathological features. (b) EMT inactive subtype's somatic mutation features. (c) EMT active subtype's somatic mutation features. (d) Comparison of two subtypes' EMT activity. (e) Comparison of two subtypes' patient prognosis. ^*∗*^*P* < 0.05, ^*∗∗*^*P* < 0.01, and ^*∗∗∗*^*P* < 0.001.

A total of 18 EMT regulators acquired form published literature were analyzed in this study. Detailed interaction patterns and coexpression states of the EMT regulators are represented in Figure S1. Given the critical role of EMT in UVM, EMT inactive subtype and EMT active subtype were identified according to the EMT regulators based on the *K*-means clustering analysis. The expression level of most EMT regulators was significantly higher in the EMT active subtype (Figure 1(a)). Somatic mutation landscape analysis of the two UVM subtypes was also performed. GNA Q had the highest mutation frequency in the EMT inactive subtype (Figure 1(b)). GNA11 was the most frequent mutation gene in the EMT active subtype (Figure 1(c)). The samples' EMT activity was estimated to further validate the accuracy of subtype identification. Sample EMT scores were significantly higher in the EMT active subtype, which confirmed the EMT active subtype's high-EMT activity (Figure 1(d), *P*=1.5*e* − 08). The Kaplan–Meier survival analysis demonstrated that the EMT active subtype had a significantly worse prognosis (Figure 1(e), *P*=8.4*e* − 05).

### 3.2. Characterization of Immune Microenvironment in the Two Subtypes

**Figure 2:**
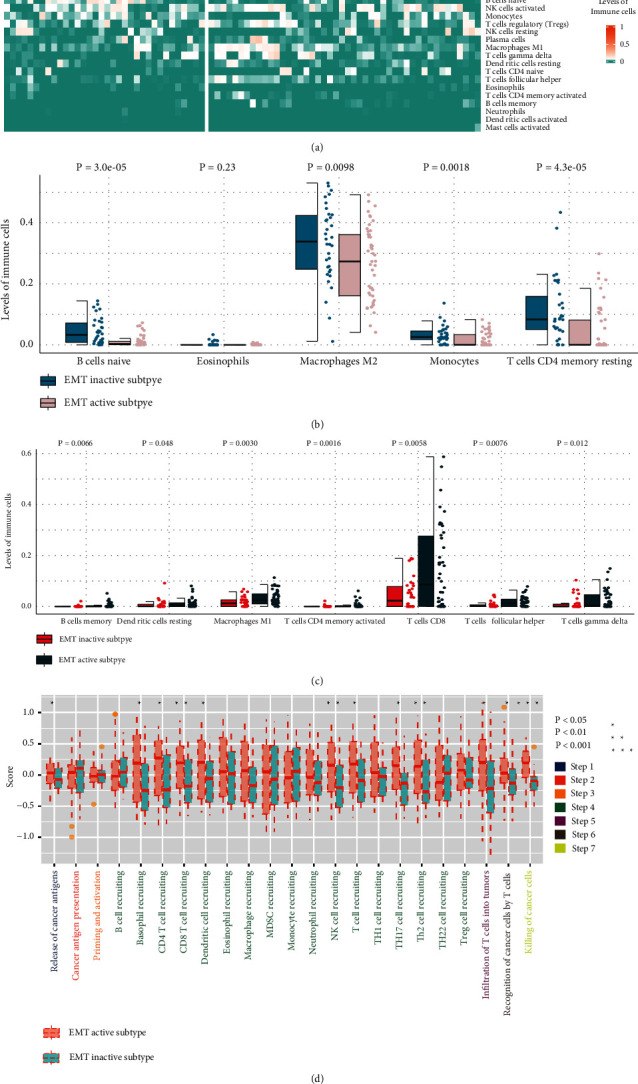
Comparison of immune characteristics of the UVM's EMT inactive subtype and EMT active subtype. (a) Subtypes' immune infiltration level was calculated using CIBERSHOT. (b, c) Comparison of two subtypes' immune infiltration levels. (d) Comparison of two subtypes' cancer immunity-related pathway activities.

To further investigate the two subtypes' microenvironment patterns, the immune cells' infiltration levels of the two subtypes was calculated (Figure 2(a)). In the EMT inactive subtypes, “T cells CD4 memory resting,” “B cells naive,” and “monocytes” had significantly higher infiltration levels (Figure 2(b)). The infiltration levels of “macrophages M1,” “T cells CD4 memory activated,” and “T cells CD8” were upregulated in the active EMT subtype (Figure 2(c)). The results implied that the EMT active subtype may have a higher proinflammatory immune response activity.

To elucidate the two subtypes' immune heterogeneity in detail, the two subtypes' immunity-circle-related pathway activities were compared. The EMT active subtype had a significantly higher activity of Step 1 (release of cancer cell antigens), Step 4 (trafficking of immune cells to tumors), Step 5 (infiltration of immune cells into tumors), Step 6 (recognition of cancer cells by T cells), and Step 7 (killing of cancer cells) (Figure 2(d)). It is worth noting that in the “trafficking of immune cells to tumors” process, the active EMT subtype's activity of CD4 and CD8 T cell recruiting was significantly higher, which is consistent with the results of the immune infiltration analysis (Figure 2(d)).

### 3.3. Construction and Validation of the EMT Feature-Based Prognostic Model

**Figure 3:**
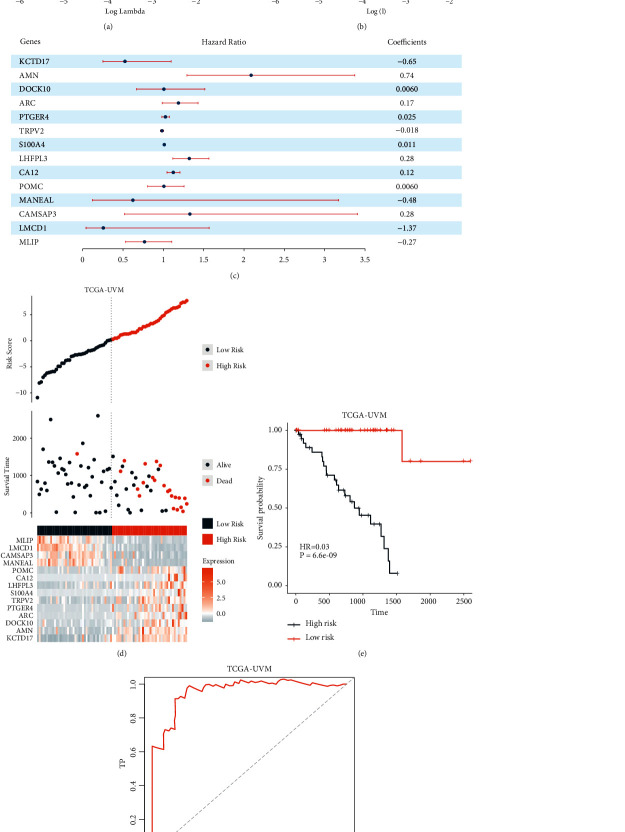
Construction of the EMT score using a machine-learning-based method. (a, b) Gene prognostic model was constructed using LASSO Cox regression. (c) Regression coefficients of model component genes. (d) EMT score component genes' expression status in TCGA-UVM cohort patients. (e) Comparison of patient prognosis in high- and low-EMT score groups from the TCGA-UVM cohort. (f) EMT score ROC curve in the TCGA-UVM cohort.

An EMT feature-based prognostic model (EMT score) was constructed using a machine-learning-based method. First, the DEGs between the EMT inactive and active subtypes were acquired. A total of 317 genes were identified (Figure S2A). Enrichment analysis of the DEGs also indicated that the two subtypes had a distinct immune microenvironment pattern (Figures S2B and S2C). Next, univariate Cox regression was employed to identify the patient outcome-related genes. A total of 117 genes were identified and submitted for the subsequent analysis (Table S2). Finally, LASSO Cox regression was used to identify the most robust prognostic genes among them and to calculate the coefficients of the selected genes (Figures 3(a) and 3(b)). The EMT feature-based gene prognostic model was described as follows:

(1) $$\begin{array}{c} \text{EMT score}=\sum_{a}\text{Coefficient}\left({\text{Gene}}_{\text{a}}\right)*\text{Expression level}\left({\text{Gene}}_{\text{a}}\right). \end{array}$$

Selected genes and their coefficients are presented in Figure 3(c).

**Figure 4:**
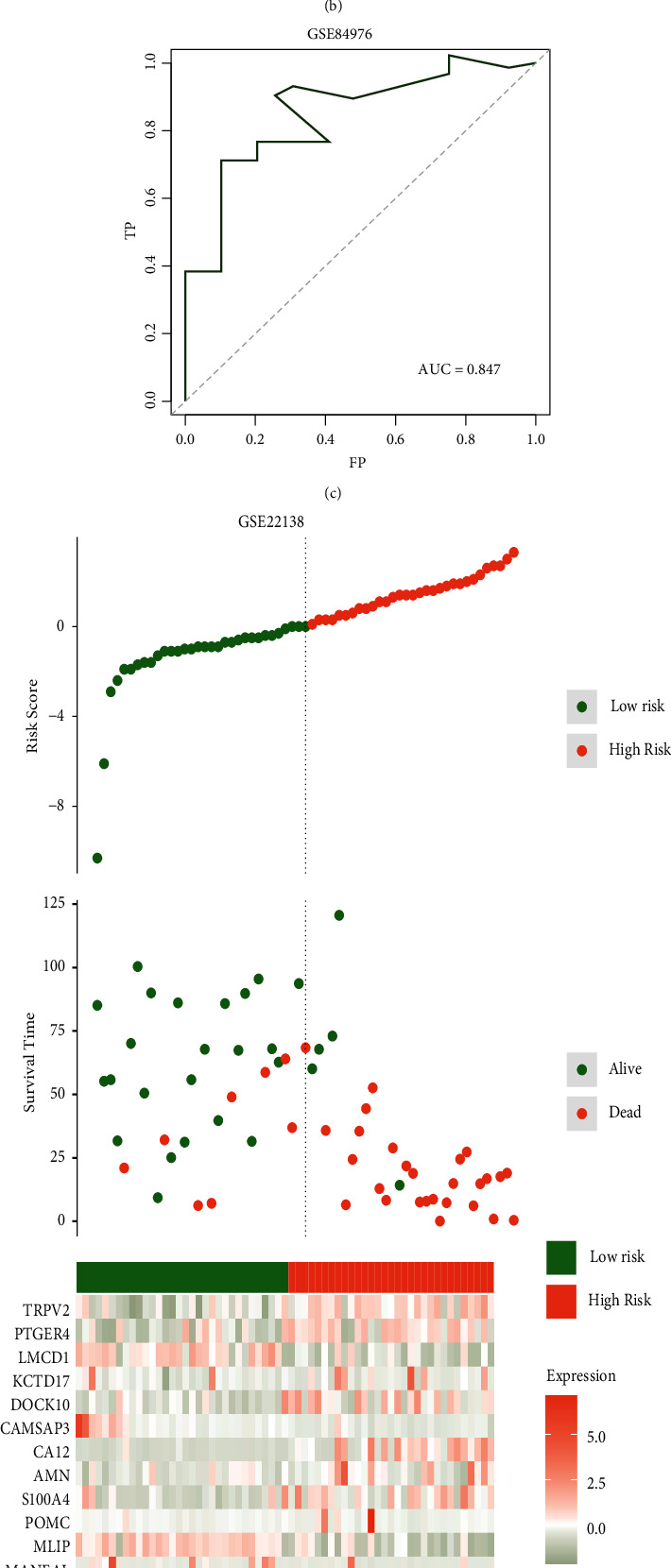
Validation of EMT score robustness in external validation sets. (a) EMT score component genes' expression status in patients from the GSE84976 cohort. (b) Comparison of patient prognosis in high- and low-EMT score groups from the GSE84976 cohort. (c) EMT score's ROC curve in the GSE84976 cohort. (d) EMT score component genes' expression status in patients from the GSE22138 cohort. (e) Comparison of patient prognosis in high- and low-EMT score groups in the GSE22138 cohort. (f) EMT score's ROC curve in the GSE22138 cohort. (g) Comparison of patient's ESTIMATE scores in high- and low-EMT score groups from the GSE84976 cohort. (h) Comparison of patient's ESTIMATE scores in high- and low-EMT score groups from the GSE22138 cohort.

To validate the robustness of the EMT score, patient outcomes in the high- and low-EMT score groups were compared. Results indicated that patients in the high-EMT score group had a significantly poorer prognosis (Figures 3(d), 3(e), *P*=6.6*e* − 09, and Figure 3(f), AUC = 0.958). Then, the correlation between the EMT score and clinical pathology characteristics was analyzed. The high-EMT score sample was more likely to be in the advanced-stage cancer (Figures S3A–S3C). The external validation cohorts GSE84976 and GSE22138 were also included to further test the EMT score's accuracy. According to the results, the model had a great prognostic value in the two validation cohorts. A high-EMT score predicted poor prognosis in UVM patients (Figures 4(a), 4(b), *P*=3.9*e* − 04, Figure 4(c), AUC = 0.847, Figure 4(d), Figure 4(e), *P* < 0.0001, and Figure 4(f), AUC = 0.75). Then, in order to explore the EMT score's potential in reflecting the tumor's immune features, the immune score of the high- and low-EMT score groups was calculated in the two external validation cohorts. The high-EMT score group had a significantly higher immune score, stromal score, and ESTIMATE score in the two cohorts, which indicated that the high-EMT score group had a significantly higher immune activity (Figures 4(g) and 4(h)).

### 3.4. Exploration of a Potential Therapy Strategy Targeting the High-EMT Score Tumor

**Figure 5:**
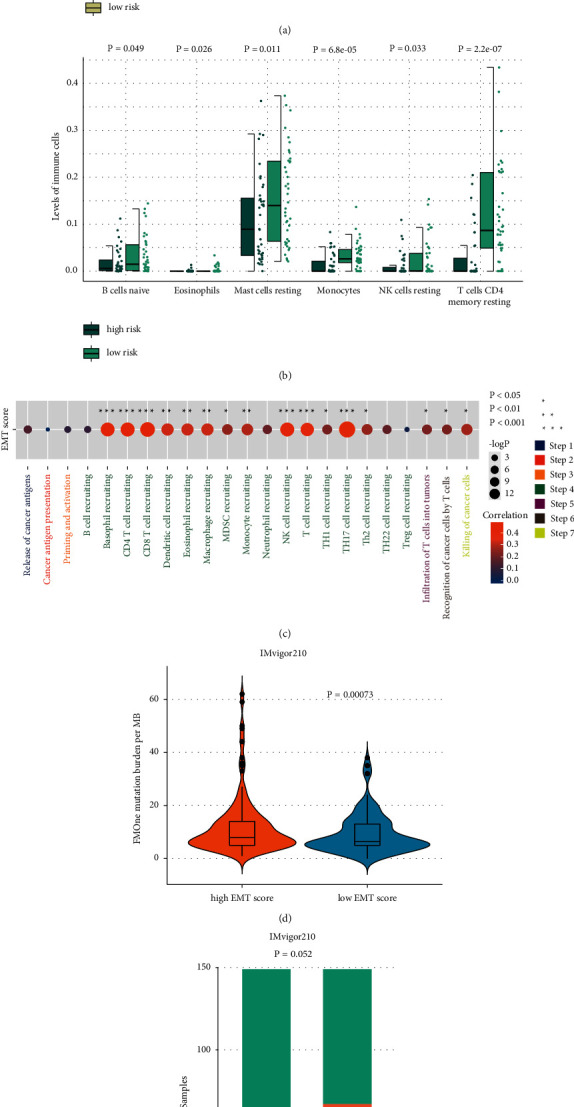
Exploration of EMT score's potential in predicting immunotherapy responsiveness. (a, b) Comparison of immune infiltration level between high- and low-EMT score groups. (c) Correlation analysis between EMT score and activity of the cancer immune-related pathway. (d) Comparison of tumor mutation burden in high- and low-EMT score groups. (e) Comparison of immunotherapy responsiveness rate in high- and low-EMT score groups. PD, progressive disease; SD, stable disease; PR, partial response; CR, complete response.

To further explore the potential therapy strategy targeting EMT active UVM, the UVM sample EMT scores were calculated according to the model and divided into high- and low-EMT score groups. According to the immune infiltration analysis, the high-EMT score group had a significantly higher infiltration level of “macrophages M1,” “T cells CD4 memory activated,” “T cells CD8,” “T cells follicular helper,” and “T cells gamma delta,” which indicated the high-EMT score's potential in reflecting a proinflammatory immune response (Figure 5(a)). On the contrary, a low-EMT score predicted the resting-like immune response pattern (Figure 5(b)). To further explore the EMT score's potential in elucidating the immune characteristics of the UVM's immunity circle, the correlation between the EMT score and the activity of the cancer-immunitycircle-related pathway was analyzed. The EMT score was able to reflect the activity of the immune cell-recruiting and cancer cell-killing process rather than the cancer-immunity circle's initial process (Figure 5(c)).

To validate the model's efficiency in predicting immunotherapy responsiveness, the immunotherapy cohort Imvigor210 was employed for subsequent analysis. In the high-EMT score group, samples had a significantly higher tumor mutation burden, which inferred the high-EMT score group's potentially high immunotherapy response rate (Figure 5(d)). To validate our hypothesis, the immunotherapy response rate of the high-EMT score group was compared to that of the low-EMT score group. The high-EMT score group had a relatively higher frequency of PR and CR (Figure 5(e)). These results demonstrated that the EMT score had great potential in predicting cancer immunotherapy responsiveness.

## 4. Discussion

UVM is a malignancy with a relatively low incidence rate but poor prognosis [4]. Although surgery and radiotherapy are effective treatment strategies for primary tumors, therapy options are limited once UVM becomes metastatic [31, 32]. Recent research has demonstrated that EMT plays an essential role in promoting UVM metastasis and contributing to the disease's poor prognosis [33–35]. However, systematic analysis to illustrate EMT-mediated tumor heterogeneity in UVM is still lacking. Robust biomarkers based on EMT features to reflect UVM's aggressiveness are also limited. Therefore, in this study, we integrated multiomics data to develop an EMT feature-based prognostic model and systematically analyzed the EMT-mediated immune microenvironment in UVM. First, EMT inactive and active subtypes were identified in UVM according to the EMT signatures acquired from multiple published sources. Somatic mutation in a GNA family gene, such as GNA11 and GNAQ, which encodes guanine nucleotide-binding protein G*α* subunits of the G*α*q family, is the driver of UVM initiation [36, 37]. GNAQ had the highest mutation frequency in the EMT inactive subtype, while GNA11 had the highest mutation frequency in the EMT active subtype. These results implied the potentially different therapy targets for EMT inactive and active UVM. Then, DEGs between the two subtypes were acquired and patient outcome-related genes were obtained using univariate Cox regression. Next, the EMT feature-based gene prognostic model was trained using patient outcome-related genes. Finally, the model's efficiency in predicting patient outcomes and therapy responsiveness was verified. This research may assist doctors in evaluating patient prognosis and choosing suitable therapy strategies in clinical practice.

The eye is considered an immune-privileged organ with partial or even completely suppressed immune responses [38]. While the concentration of antitumor immune cells in the microenvironment is correlated with a better outcome in most cancer types, the immune infiltration in UVM can direct to poor prognosis [39]. Emerging studies have emphasized the strong interaction between EMT and tumor immune microenvironment [40]. Here, we analyzed the immune microenvironment characteristics of EMT inactive and active subtypes. The results demonstrated that EMT may result in the high infiltration and high activity of proinflammatory immune cells. EMT plays a crucial role in tumor microenvironment progression. For example, EMT transcriptional factors, including Snail, Zeb1, and Twist1, can attract cancer-related immune cells and shape tumor microenvironment into a protumor subtype [41, 42]. In turn, the modulated microenvironment can promote cancer EMT [43, 44]. Thus, therapy strategies that can interfere with this positive feedback system may introduce clinical benefits to the EMT active UVM subtype. When comparing the two groups' antitumor immune process activities, we found that the EMT active subtype's active anticancer-immune-related pathways were concentrated on the immune cells recruiting the related process and “infiltration of T cells into tumors,” “recognition of cancer cells by T cells,” and “killing of cancer cells” pathway. These EMT-mediated immune features may be the potential underlying mechanism for EMT-mediated UVM's poor prognosis. This EMT-mediated immune characteristic also suggested that a therapy targeting these immune processes may be suitable for the EMT active UVM.

UVM has a high metastatic rate, and clinical outcomes for metastatic UVM are unsatisfactory [45]. While the nonmetastatic UVM has a relatively good prognosis, once distant UVM metastases have occurred, the clinical treatment strategy will be limited [2]. Thus, apart from the traditional pathological detection methods, a supplementary method to assess the UVM's clinical pathology features is important. It was found that the tumor EMT score was significantly higher in advanced UVM (T4, M1, and stage IV). The EMT score also had great efficiency in evaluating UVM prognosis and immune activity. Therefore, the EMT score may be developed as a novel biomarker to predict UVM prognosis, metastasis status, and immune features.

The cancer-immunity cycle reflects the immune response of the inherent and adaptive immune systems to UVM. The goal of cancer immunotherapy is to initiate or reinitiate a self-sustaining cycle of cancer immunity to help the immune system conduct the cytotoxic tumor-killing process [46]. Every step of the cancer-immunity circle plays an essential role in immunotherapy response. Cancer immunotherapy strategies need to be developed based on the features of the cancer-immunity circle. For example, a cancer vaccine would be suitable for Step 1 (release of cancer antigens) dysfunction cancer subtype [47]. The activities of the cancer-immunitycycle-related pathways reflect the comprehensive immunomodulatory interactions in UVM tumor microenvironment. The present study explored the EMT score's potential in predicting the activity of the pathways. According to the results, the EMT score was able to reflect the activity of the immune cell recruiting process and tumor cytotoxic killing-related processes, while the correlation between the EMT score and activity of tumor antigen-based immune cell activation-related processes was not observed. A novel therapy strategy targeting these immunity-related pathways might introduce clinical benefits to UVM patients with high-EMT scores.

Cancer immunotherapy, which targets the tumor immune escape mechanisms and activates the body's immune system to recognize and attack cancer cells, has become the emerging strategy for comprehensive cancer treatments in clinical practice [48–51]. Immunotherapy has brought revolutionary progress to clinical tumor treatment. The application of immune checkpoint blockade in malignancies, including melanoma, urothelial bladder cancer, head and neck squamous cell carcinoma, and classical Hodgkin's lymphoma, has brought significant clinical benefits to patients [52–55]. However, the nonignorable nonresponsiveness rate and the related side effects in the clinical practice have been the major obstacles for its implementation [56]. In the present research, the EMT score provides a method to identify the potential candidates for UVM immunotherapy. The results indicated that patients with high-EMT scores may have the potentially high responsiveness rate for immunotherapy.

The present study had some limitations. First, the enrolled UVM and immunotherapy cohorts were limited. More clinical information and transcriptome data should be utilized to validate the EMT score efficiency. Second, although the EMT-mediated immune microenvironment alteration was analyzed, the detailed underlying immune regulatory network remains to be further elucidated. Third, the EMT score's robustness should be further tested based on clinical trials. Related research would be important for the EMT score's clinical application. These shortcomings will be alleviated with the development of large data pools and further research.

## 5. Conclusions

In summary, we developed an EMT score to predict UVM patient prognosis, immune microenvironment characteristics, and immunotherapy responsiveness in a machine-learning-based method. The EMT score robustness was validated by two external validation cohorts. The EMT score predicted the UVM patient outcomes and immune activity in the training and validation cohorts. The immunotherapy cohort-based analysis revealed the EMT score's potential in the preliminary identification of immunotherapy candidates. This research may facilitate precise treatment in a further clinic-integrated oncology therapy of UVM.

## Data Availability

The microarray data supporting this study are from previously reported databases, which have been cited. The processed data are available from the corresponding author upon request.

## Supplementary Materials

Supplementary Materials Figure S1: EMT signature interaction status, coexpression status, and PPI network. Figure S2: differential expression gene acquisition and enrichment analysis. Figure S3: analysis of correlation between clinicopathological features and EMT score. Table S1: cancer-immunity circle pathway. Table S2: Cox regression analysis for genes.
