# Supplementary material for: EMT-Related Gene Signature Predicts the Prognosis in Uveal Melanoma Patients
Source: J Oncol. 2022 Aug 12;2022:5436988. doi: 10.1155/2022/5436988 (PMC9391141; doi:10.1155/2022/5436988)
Supplement: Supplementary Materials — Figure S1: EMT signature interaction status, coexpression status, and PPI network. Figure S2: differential expression gene acquisition and enrichment analysis. Figure S3: analysis of correlation between clinicopathological features and EMT score. Table S1: cancer-immunity circle pathway. Table S2: Cox regression analysis for genes. [file 5436988.f1.docx]

## Supplementary Materials


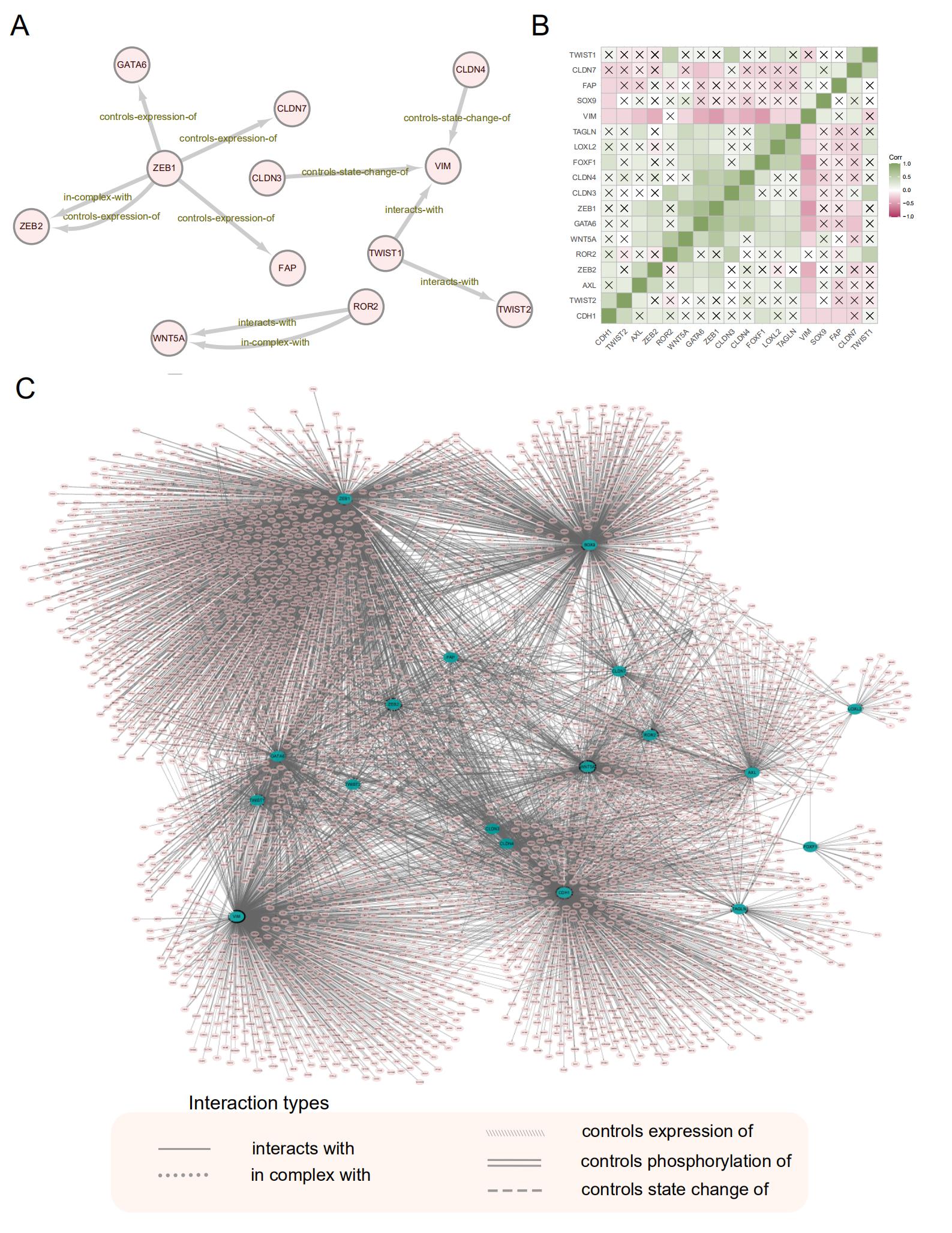


Figure S1. EMT signature interaction status, co-expression status, and PPI network.

(A): EMT signature interaction status. (B): EMT signature co-expression status. (C): EMT signature PPI network.


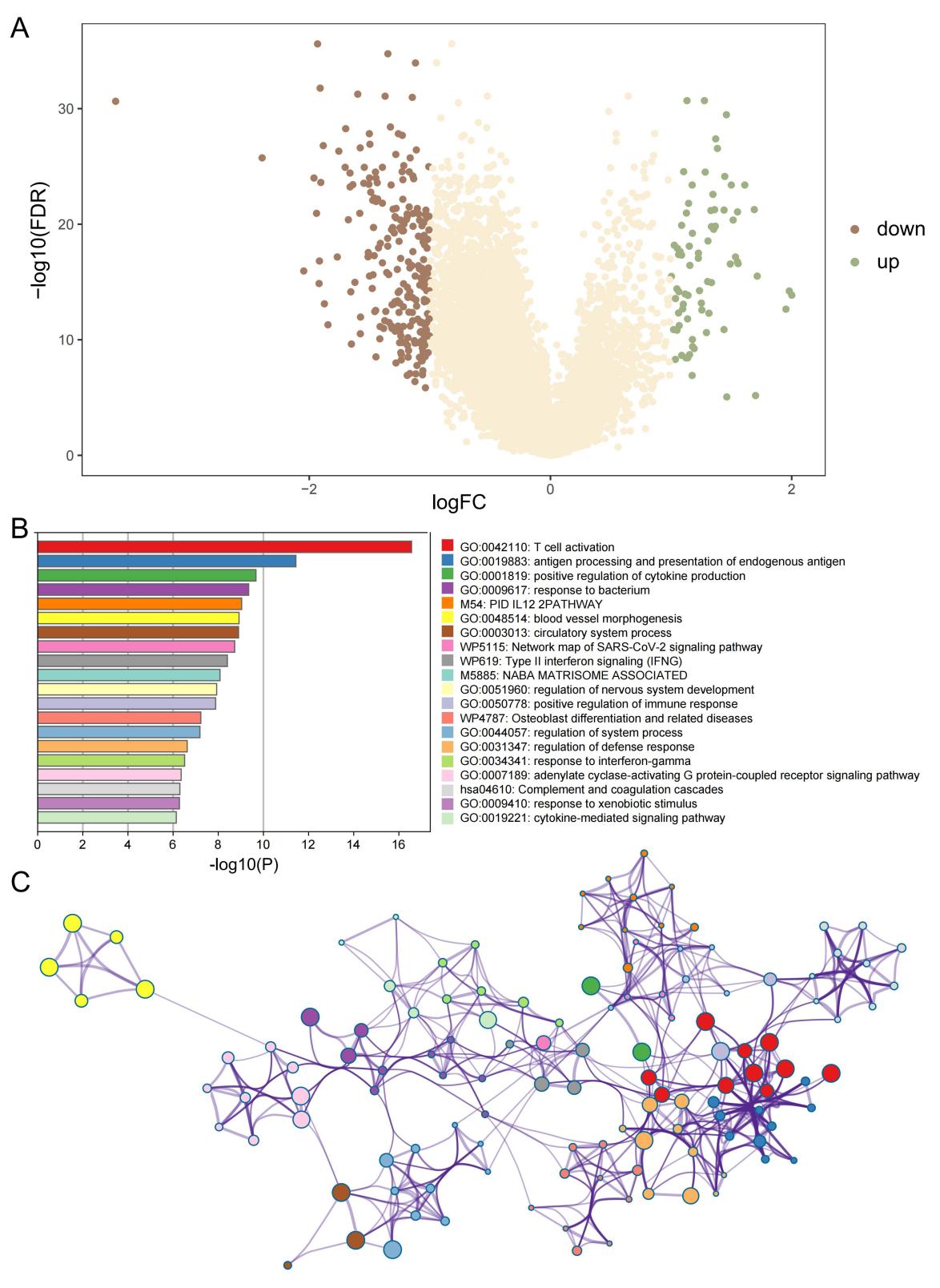


FigureS2. Differential expression genes’ acquisition and enrichment analysis.

(A): Volcano map of differential expression genes. (B-C): Enrichment analysis of differential expression genes.


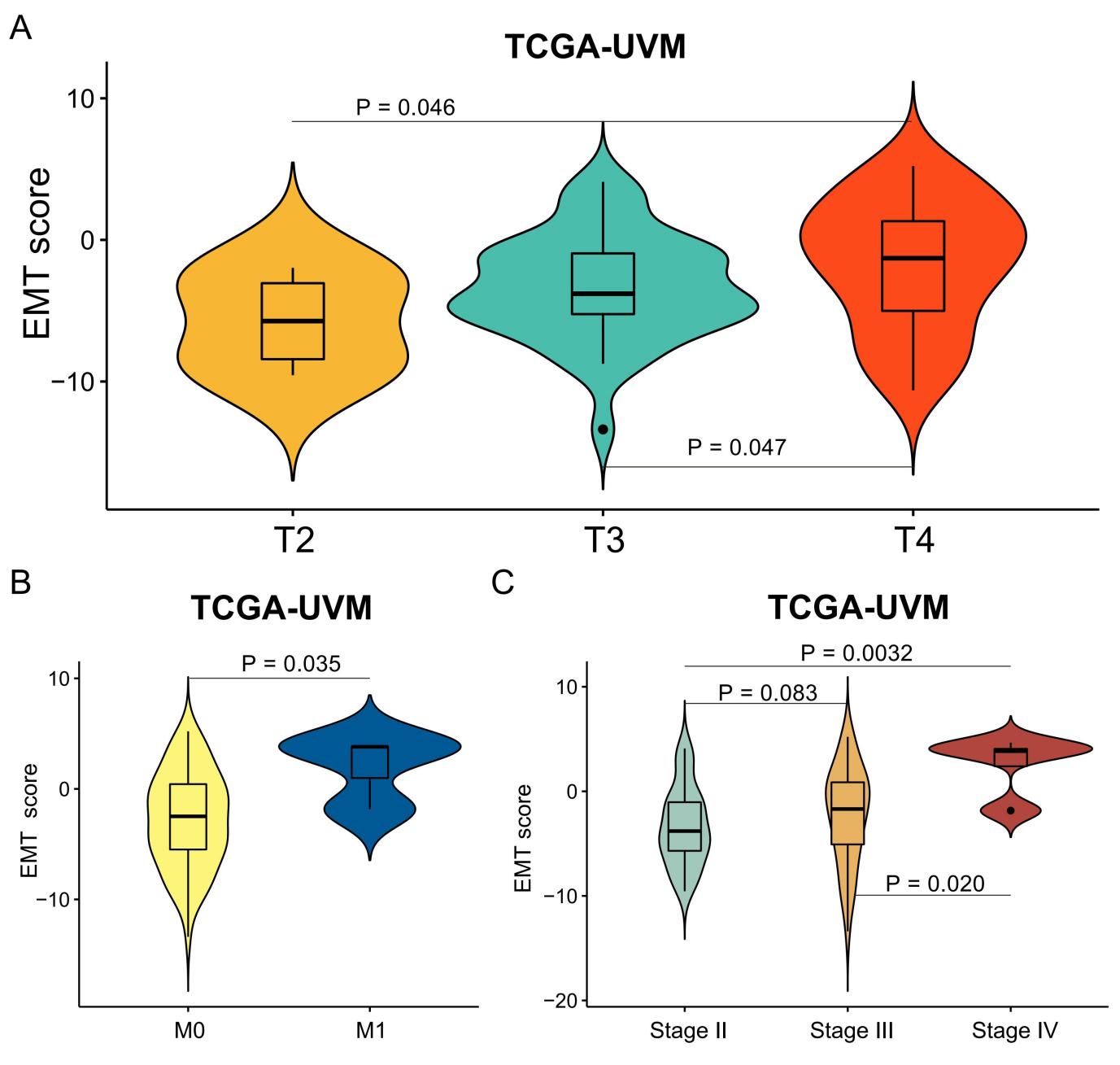


FigureS3. Analysis of the correlation between the clinicopathological features and EMT score.

(A): Comparison of sample’s EMT score with different T status. (B): Comparison of sample’s EMT score with different M status. (C): Comparison of sample’s EMT score with different stage status.

**Table S1 Cancer immune circle pathway**

| Steps | pathway | gene-  promote | gene-inhibit |
| --- | --- | --- | --- |
| 1. Release of cancer antigens | N/A | IL10 |  |
| 1. Release of cancer antigens | N/A | TGFB1 |  |
| 1. Release of cancer antigens | N/A | HMGB1 |  |
| 1. Release of cancer antigens | N/A | ANXA1 |  |
| 1. Release of cancer antigens | N/A | CALR |  |
| 1. Release of cancer antigens | N/A | CXCL10 |  |
| 1. Release of cancer antigens | N/A | PDIA3 |  |
| 1. Release of cancer antigens | N/A | HSPA1A |  |
| 1. Release of cancer antigens | N/A | HSPA1B |  |
| 1. Release of cancer antigens | N/A | HSPA2 |  |
| 1. Release of cancer antigens | N/A | HSPA8 |  |
| 1. Release of cancer antigens | N/A | HSPA4 |  |
| 1. Release of cancer antigens | N/A | HSPA14 |  |
| 1. Release of cancer antigens | N/A | HSPA5 |  |
| 1. Release of cancer antigens | N/A | HSPA6 |  |
| 1. Release of cancer antigens | N/A | HSPA9 |  |
| 1. Release of cancer antigens | N/A | HSPA13 |  |
| 1. Release of cancer antigens | N/A | HSPA7 |  |
| 1. Release of cancer antigens | N/A | HSPA8 |  |
| 1. Release of cancer antigens | N/A | HSPA12A |  |
| 1. Release of cancer antigens | N/A | HSPA12B |  |
| 1. Release of cancer antigens | N/A | HSP90AA1 |  |
| 1. Release of cancer antigens | N/A | HSP90AB1 |  |
| 1. Release of cancer antigens | N/A | HSP90B1 |  |
| 1. Release of cancer antigens | N/A | IFNA2 |  |
| 1. Release of cancer antigens | N/A | IFNA1 |  |
| 1. Release of cancer antigens | N/A | IFNA13 |  |
| 1. Release of cancer antigens | N/A | IFNA6 |  |
| 1. Release of cancer antigens | N/A | IFNA21 |  |
| 1. Release of cancer antigens | N/A | IFNA4 |  |
| 1. Release of cancer antigens | N/A | IFNA8 |  |
| 1. Release of cancer antigens | N/A | IFNA5 |  |
| 1. Release of cancer antigens | N/A | IFNA7 |  |
| 1. Release of cancer antigens | N/A | IFNA14 |  |
| 1. Release of cancer antigens | N/A | IFNA16 |  |
| 1. Release of cancer antigens | N/A | IFNA10 |  |
| 1. Release of cancer antigens | N/A | IFNA17 |  |
| 1. Release of cancer antigens | N/A | IFNB1 |  |
| 1. Release of cancer antigens | N/A | IFNE |  |
| 1. Release of cancer antigens | N/A | IFNW1 |  |
| 2. Cancer antigen presentation | N/A | IL1B | IL10 |
| 2. Cancer antigen presentation | N/A | IFNA2 | IL4 |
| 2. Cancer antigen presentation | N/A | IFNA1 | IL13 |
| 2. Cancer antigen presentation | N/A | IFNA13 |  |
| 2. Cancer antigen presentation | N/A | IFNA6 |  |
| 2. Cancer antigen presentation | N/A | IFNA21 |  |
| 2. Cancer antigen presentation | N/A | IFNA4 |  |
| 2. Cancer antigen presentation | N/A | IFNA8 |  |
| 2. Cancer antigen presentation | N/A | IFNA5 |  |
| 2. Cancer antigen presentation | N/A | IFNA7 |  |
| 2. Cancer antigen presentation | N/A | IFNA14 |  |
| 2. Cancer antigen presentation | N/A | IFNA16 |  |
| 2. Cancer antigen presentation | N/A | IFNA10 |  |
| 2. Cancer antigen presentation | N/A | IFNA17 |  |
| 2. Cancer antigen presentation | N/A | CD40LG |  |
| 2. Cancer antigen presentation | N/A | CD40 |  |
| 2. Cancer antigen presentation | N/A | NT5C |  |
| 2. Cancer antigen presentation | N/A | HMGB1 |  |
| 2. Cancer antigen presentation | N/A | TLR1 |  |
| 2. Cancer antigen presentation | N/A | TLR2 |  |
| 2. Cancer antigen presentation | N/A | TLR3 |  |
| 2. Cancer antigen presentation | N/A | TLR4 |  |
| 2. Cancer antigen presentation | N/A | TLR5 |  |
| 2. Cancer antigen presentation | N/A | TLR6 |  |
| 2. Cancer antigen presentation | N/A | TLR7 |  |
| 2. Cancer antigen presentation | N/A | TLR8 |  |
| 2. Cancer antigen presentation | N/A | TLR9 |  |
| 2. Cancer antigen presentation | N/A | TLR10 |  |
| 2. Cancer antigen presentation | N/A | HLAA |  |
| 2. Cancer antigen presentation | N/A | B2M |  |
| 2. Cancer antigen presentation | N/A | TAP1 |  |
| 3. Priming and activation | Multiple | CD3D | CTLA4 |
| 3. Priming and activation | Multiple | CD3E | PDCD1 |
| 3. Priming and activation | Multiple | CD3G | PDCD1LG2 |
| 3. Priming and activation | Multiple | CD247 | CD274 |
| 3. Priming and activation | Multiple | CD28 | CD160 |
| 3. Priming and activation | Multiple | TNFRSF9 | TNFRSF14 |
| 3. Priming and activation | Multiple | TNFSF9 | BTLA |
| 3. Priming and activation | Multiple | TNFRSF4 | VSIR |
| 3. Priming and activation | Multiple | TNFSF4 | LAIR1 |
| 3. Priming and activation | Multiple | CD27 | HAVCR1 |
| 3. Priming and activation | Multiple | CD70 | HAVCR2 |
| 3. Priming and activation | Multiple | TNFRSF14 | LGALS9 |
| 3. Priming and activation | Multiple | TNFSF14 | TIMD4 |
| 3. Priming and activation | Multiple | CD40 | CD244 |
| 3. Priming and activation | Multiple | CD40LG | CD48 |
| 3. Priming and activation | Multiple | TNFRSF18 | TIGIT |
| 3. Priming and activation | Multiple | TNFSF18 | NECTIN3 |
| 3. Priming and activation | Multiple | TNFRSF25 | LAG3 |
| 3. Priming and activation | Multiple | TNFSF15 |  |
| 3. Priming and activation | Multiple | TNFRSF8 |  |
| 3. Priming and activation | Multiple | TNFSF8 |  |
| 3. Priming and activation | Multiple | HAVCR1 |  |
| 3. Priming and activation | Multiple | TIMD4 |  |
| 3. Priming and activation | Multiple | SLAMF7 |  |
| 3. Priming and activation | Multiple | SLAMF6 |  |
| 3. Priming and activation | Multiple | SLAMF1 |  |
| 3. Priming and activation | Multiple | SLAMF9 |  |
| 3. Priming and activation | Multiple | SLAMF8 |  |
| 3. Priming and activation | Multiple | CD2 |  |
| 3. Priming and activation | Multiple | CD48 |  |
| 3. Priming and activation | Multiple | CD58 |  |
| 3. Priming and activation | Multiple | CD226 |  |
| 3. Priming and activation | Multiple | ICOS |  |
| 3. Priming and activation | Multiple | ICOSLG |  |
| 3. Priming and activation | Multiple | KLRK1 |  |
| 3. Priming and activation | Multiple | MICA |  |
| 3. Priming and activation | Multiple | MICB |  |
| 3. Priming and activation | Multiple | RAET1E |  |
| 3. Priming and activation | Multiple | RAET1G |  |
| 3. Priming and activation | Multiple | CRTAM |  |
| 3. Priming and activation | Multiple | CADM1 |  |
| 3. Priming and activation | Multiple | IL2 |  |
| 3. Priming and activation | Multiple | IL12A |  |
| 3. Priming and activation | Multiple | IL12B |  |
| 4. Trafficking of T cells to tumors | B cell recruiting | CXCR5 |  |
| 4. Trafficking of T cells to tumors | B cell recruiting | CXCL13 |  |
| 4. Trafficking of T cells to tumors | Basophil recruiting | CCL24 |  |
| 4. Trafficking of T cells to tumors | Basophil recruiting | CCL26 |  |
| 4. Trafficking of T cells to tumors | CD4 T cell recruiting | CCL19 |  |
| 4. Trafficking of T cells to tumors | CD4 T cell recruiting | CX3CL1 |  |
| 4. Trafficking of T cells to tumors | CD4 T cell recruiting | CXCL16 |  |
| 4. Trafficking of T cells to tumors | CD8 T cell recruiting | CCR5 |  |
| 4. Trafficking of T cells to tumors | CD8 T cell recruiting | CXCR3 |  |
| 4. Trafficking of T cells to tumors | CD8 T cell recruiting | CXCL10 |  |
| 4. Trafficking of T cells to tumors | CD8 T cell recruiting | CXCL9 |  |
| 4. Trafficking of T cells to tumors | CD8 T cell recruiting | CCL20 |  |
| 4. Trafficking of T cells to tumors | CD8 T cell recruiting | CXCL11 |  |
| 4. Trafficking of T cells to tumors | CD8 T cell recruiting | CX3CL1 |  |
| 4. Trafficking of T cells to tumors | CD8 T cell recruiting | CXCL16 |  |
| 4. Trafficking of T cells to tumors | Dendritic cell recruiting | CCR7 |  |
| 4. Trafficking of T cells to tumors | Dendritic cell recruiting | CCL3 |  |
| 4. Trafficking of T cells to tumors | Dendritic cell recruiting | CCL4 |  |
| 4. Trafficking of T cells to tumors | Dendritic cell recruiting | CCL5 |  |
| 4. Trafficking of T cells to tumors | Dendritic cell recruiting | CCL21 |  |
| 4. Trafficking of T cells to tumors | Eosinophil recruiting | CCL11 |  |
| 4. Trafficking of T cells to tumors | Eosinophil recruiting | CCL24 |  |
| 4. Trafficking of T cells to tumors | Eosinophil recruiting | CCL26 |  |
| 4. Trafficking of T cells to tumors | Macrophage recruiting | CSF1 |  |
| 4. Trafficking of T cells to tumors | Macrophage recruiting | CCL2 |  |
| 4. Trafficking of T cells to tumors | Macrophage recruiting | CCL3 |  |
| 4. Trafficking of T cells to tumors | Macrophage recruiting | CCL4 |  |
| 4. Trafficking of T cells to tumors | Macrophage recruiting | CCL5 |  |
| 4. Trafficking of T cells to tumors | MDSC recruiting | CXCR2 |  |
| 4. Trafficking of T cells to tumors | MDSC recruiting | CXCL5 |  |
| 4. Trafficking of T cells to tumors | Monocyte recruiting | CCL2 |  |
| 4. Trafficking of T cells to tumors | Monocyte recruiting | CCL7 |  |
| 4. Trafficking of T cells to tumors | Monocyte recruiting | CX3CL1 |  |
| 4. Trafficking of T cells to tumors | Neutrophil recruiting | CXCL1 |  |
| 4. Trafficking of T cells to tumors | Neutrophil recruiting | CXCL2 |  |
| 4. Trafficking of T cells to tumors | Neutrophil recruiting | CXCL3 |  |
| 4. Trafficking of T cells to tumors | Neutrophil recruiting | CXCL8 |  |
| 4. Trafficking of T cells to tumors | Neutrophil recruiting | CXCL6 |  |
| 4. Trafficking of T cells to tumors | Neutrophil recruiting | CXCL5 |  |
| 4. Trafficking of T cells to tumors | NK cell recruiting | CXCR3 |  |
| 4. Trafficking of T cells to tumors | NK cell recruiting | CXCL10 |  |
| 4. Trafficking of T cells to tumors | NK cell recruiting | CXCL9 |  |
| 4. Trafficking of T cells to tumors | NK cell recruiting | CCL3 |  |
| 4. Trafficking of T cells to tumors | NK cell recruiting | CCL4 |  |
| 4. Trafficking of T cells to tumors | NK cell recruiting | CCL5 |  |
| 4. Trafficking of T cells to tumors | NK cell recruiting | CXCL11 |  |
| 4. Trafficking of T cells to tumors | NK cell recruiting | CX3CL1 |  |
| 4. Trafficking of T cells to tumors | T cell recruiting | CXCR5 |  |
| 4. Trafficking of T cells to tumors | T cell recruiting | CCR7 |  |
| 4. Trafficking of T cells to tumors | T cell recruiting | CXCL9 |  |
| 4. Trafficking of T cells to tumors | T cell recruiting | CCL3 |  |
| 4. Trafficking of T cells to tumors | T cell recruiting | CCL4 |  |
| 4. Trafficking of T cells to tumors | T cell recruiting | CCL5 |  |
| 4. Trafficking of T cells to tumors | T cell recruiting | CCL19 |  |
| 4. Trafficking of T cells to tumors | T cell recruiting | CCL21 |  |
| 4. Trafficking of T cells to tumors | T cell recruiting | CX3CL1 |  |
| 4. Trafficking of T cells to tumors | T cell recruiting | CXCL13 |  |
| 4. Trafficking of T cells to tumors | TH1 cell recruiting | CXCR3 |  |
| 4. Trafficking of T cells to tumors | TH1 cell recruiting | CXCL10 |  |
| 4. Trafficking of T cells to tumors | TH1 cell recruiting | CXCL9 |  |
| 4. Trafficking of T cells to tumors | TH1 cell recruiting | CXCL11 |  |
| 4. Trafficking of T cells to tumors | TH17 cell recruiting | CCR6 |  |
| 4. Trafficking of T cells to tumors | TH17 cell recruiting | CCL20 |  |
| 4. Trafficking of T cells to tumors | TH17 cell recruiting | CXCL12 |  |
| 4. Trafficking of T cells to tumors | TH17 cell recruiting | CXCR4 |  |
| 4. Trafficking of T cells to tumors | Th2 cell recruiting | CCL1 |  |
| 4. Trafficking of T cells to tumors | Th2 cell recruiting | CCL17 |  |
| 4. Trafficking of T cells to tumors | Th2 cell recruiting | CCL22 |  |
| 4. Trafficking of T cells to tumors | TH22 cell recruiting | CCR6 |  |
| 4. Trafficking of T cells to tumors | TH22 cell recruiting | CCL20 |  |
| 4. Trafficking of T cells to tumors | Treg cell recruiting | CCR4 |  |
| 4. Trafficking of T cells to tumors | Treg cell recruiting | CCR10 |  |
| 4. Trafficking of T cells to tumors | Treg cell recruiting | CCL1 |  |
| 4. Trafficking of T cells to tumors | Treg cell recruiting | CCL17 |  |
| 4. Trafficking of T cells to tumors | Treg cell recruiting | CCL22 |  |
| 4. Trafficking of T cells to tumors | Treg cell recruiting | CCL28 |  |
| 5. Infiltration of T cells into tumors | N/A | STAT1 | ICAM1 |
| 5. Infiltration of T cells into tumors | N/A | IRF5 | EZH2 |
| 5. Infiltration of T cells into tumors | N/A | KLF2 | DNMT1 |
| 5. Infiltration of T cells into tumors | N/A | ITGB2 | VEGFA |
| 5. Infiltration of T cells into tumors | N/A |  | EDNRB |
| 6. Recognition of cancer cells by T cells | N/A | CD28 | PDCD1 |
| 6. Recognition of cancer cells by T cells | N/A | ICOS | PDCD1LG2 |
| 6. Recognition of cancer cells by T cells | N/A | ICOSLG | CD274 |
| 6. Recognition of cancer cells by T cells | N/A | TNFRSF9 | CTLA4 |
| 6. Recognition of cancer cells by T cells | N/A | TNFSF9 | BTLA |
| 6. Recognition of cancer cells by T cells | N/A | CD27 | VTCN1 |
| 6. Recognition of cancer cells by T cells | N/A | CD70 |  |
| 6. Recognition of cancer cells by T cells | N/A | TNFRSF4 |  |
| 6. Recognition of cancer cells by T cells | N/A | TNFSF4 |  |
| 6. Recognition of cancer cells by T cells | N/A | TNFSF14 |  |
| 6. Recognition of cancer cells by T cells | N/A | CD40 |  |
| 6. Recognition of cancer cells by T cells | N/A | CD40LG |  |
| 6. Recognition of cancer cells by T cells | N/A | HLAA |  |
| 6. Recognition of cancer cells by T cells | N/A | B2M |  |
| 6. Recognition of cancer cells by T cells | N/A | TAP1 |  |
| 6. Recognition of cancer cells by T cells | N/A | BIRC5 |  |
| 6. Recognition of cancer cells by T cells | N/A | MDM2 |  |
| 6. Recognition of cancer cells by T cells | N/A | MAGEA4 |  |
| 6. Recognition of cancer cells by T cells | N/A | TP53 |  |
| 7. Killing of cancer cells | N/A | IFNG | PDCD1 |
| 7. Killing of cancer cells | N/A | GZMB | SMC3 |
| 7. Killing of cancer cells | N/A | PRF1 | VTCN1 |
| 7. Killing of cancer cells | N/A |  | HAVCR2 |
| 7. Killing of cancer cells | N/A |  | MICA |
| 7. Killing of cancer cells | N/A |  | MICB |
| 7. Killing of cancer cells | N/A |  | BTLA |
| 7. Killing of cancer cells | N/A |  | VSIR |
| 7. Killing of cancer cells | N/A |  | LAG3 |
| 7. Killing of cancer cells | N/A |  | IDO1 |
| 7. Killing of cancer cells | N/A |  | IDO2 |
| 7. Killing of cancer cells | N/A |  | ARG1 |
| 7. Killing of cancer cells | N/A |  | ARG2 |
| 7. Killing of cancer cells | N/A |  | NOS1 |
| 7. Killing of cancer cells | N/A |  | NOS2 |
| 7. Killing of cancer cells | N/A |  | NOS3 |
| 7. Killing of cancer cells | N/A |  | TGFB1 |
| 7. Killing of cancer cells | N/A |  | IL10 |
| 7. Killing of cancer cells | N/A |  | CCL28 |
| 7. Killing of cancer cells | N/A |  | CXCL12 |
| 7. Killing of cancer cells | N/A |  | CCL2 |
| 7. Killing of cancer cells | N/A |  | CXCL8 |

**Table S2 Cox regression genes**

| DEGs | COX coefficient | HR | P value |
| --- | --- | --- | --- |
| CELF2 | 0.163900266 | 1.17809681 | 5.00E-04 |
| CALHM2 | 0.323831217 | 1.38241396 | 1.24E-06 |
| SAP30 | 0.352802263 | 1.42304973 | 6.46E-04 |
| AHNAK2 | 0.082067781 | 1.08552939 | 7.30E-05 |
| DLL4 | 0.227813615 | 1.25585123 | 2.02E-05 |
| KCTD17 | 0.362524374 | 1.43695224 | 2.30E-05 |
| FADS1 | 0.261949322 | 1.29946069 | 7.61E-07 |
| HTR2B | 0.015824536 | 1.01595041 | 6.56E-05 |
| PPM1K | 0.167813551 | 1.18271607 | 1.27E-05 |
| ADAM11 | 0.259750148 | 1.29660609 | 1.17E-04 |
| RAB31 | 0.227284614 | 1.25518706 | 5.05E-06 |
| NQO1 | 0.016328551 | 1.01646259 | 1.72E-05 |
| CHAC1 | 0.011321519 | 1.01138585 | 1.37E-04 |
| SGSM2 | 0.050371223 | 1.05166142 | 5.20E-04 |
| HES6 | 0.023833231 | 1.02411951 | 1.18E-04 |
| HTRA3 | 0.315645681 | 1.37114435 | 1.01E-05 |
| FKBP11 | 0.079501837 | 1.08274755 | 5.48E-06 |
| GDF11 | 0.097374405 | 1.10227299 | 1.32E-04 |
| CARD11 | 0.211639146 | 1.2357019 | 7.71E-05 |
| MAPK12 | 0.355940554 | 1.42752269 | 1.17E-05 |
| ASB9 | 0.105131907 | 1.11085713 | 3.60E-04 |
| JPH1 | 0.632122293 | 1.88159965 | 9.45E-06 |
| TNFRSF19 | 0.114371936 | 1.12116905 | 1.84E-07 |
| LINGO1 | 0.11004055 | 1.11632334 | 1.14E-04 |
| FKBP5 | 0.382629337 | 1.46613449 | 1.47E-05 |
| ITPR2 | 0.191535485 | 1.21110781 | 1.08E-04 |
| IGFBP2 | 0.040364287 | 1.04119 | 7.77E-05 |
| PTP4A3 | 0.006766643 | 1.00678959 | 4.84E-05 |
| AMN | 0.217330156 | 1.24275434 | 6.48E-05 |
| TMEM255A | 0.184663607 | 1.20281375 | 9.48E-05 |
| IGFBP7 | 0.005529232 | 1.00554455 | 8.70E-05 |
| PLEKHG4B | 0.326493457 | 1.38609918 | 7.73E-07 |
| JAG1 | 0.286499769 | 1.33175786 | 4.64E-04 |
| CITED1 | 0.004476503 | 1.00448654 | 1.12E-06 |
| ADM2 | 0.631320703 | 1.88009198 | 5.93E-06 |
| GEM | 0.10907454 | 1.11524548 | 1.37E-04 |
| ECM1 | 0.02496055 | 1.02527467 | 7.64E-05 |
| FAM132B | 0.437091935 | 1.5481984 | 3.93E-06 |
| CEBPD | 0.069360782 | 1.07182283 | 2.89E-04 |
| FABP5 | 0.121789572 | 1.1295164 | 8.99E-06 |
| FKBP10 | 0.016967558 | 1.01711232 | 6.02E-06 |
| JAG2 | 0.188566599 | 1.2075175 | 7.79E-06 |
| ISG20 | 0.431031607 | 1.53884419 | 1.31E-07 |
| FZD7 | 0.074525657 | 1.07737299 | 3.18E-04 |
| SULF2 | 0.091613932 | 1.09594163 | 4.53E-04 |
| BAI1 | 0.197655814 | 1.21854292 | 3.47E-06 |
| DOCK10 | 0.384387617 | 1.46871463 | 2.76E-05 |
| CDC25B | 0.054793028 | 1.05632196 | 4.11E-05 |
| BAG2 | 0.344685896 | 1.41154648 | 5.31E-05 |
| SLC1A4 | 0.055432592 | 1.05699777 | 2.58E-04 |
| NFATC4 | 0.339592715 | 1.40437549 | 3.05E-04 |
| BCAT1 | 0.588646909 | 1.8015491 | 6.60E-04 |
| SLCO5A1 | 0.369930614 | 1.44763417 | 3.39E-05 |
| TGFBI | 0.106441529 | 1.11231289 | 2.45E-05 |
| LHFP | 0.133989353 | 1.14338065 | 8.84E-04 |
| SPON2 | 0.021393394 | 1.02162387 | 6.59E-07 |
| AK4 | 0.443437061 | 1.55805315 | 1.21E-04 |
| PARP8 | 0.858564404 | 2.35977058 | 1.17E-06 |
| SLC1A1 | 0.474860916 | 1.60779056 | 1.71E-04 |
| ARC | 0.286430091 | 1.33166507 | 7.56E-07 |
| PRKCDBP | 0.049338238 | 1.05057563 | 1.37E-04 |
| ARMC9 | 0.074592082 | 1.07744455 | 2.67E-05 |
| NECAB2 | 0.041910952 | 1.04280161 | 4.15E-05 |
| FERMT3 | 0.036722305 | 1.0374049 | 3.38E-05 |
| MYEOV | 0.115089664 | 1.12197403 | 7.30E-05 |
| THBS2 | 0.173584978 | 1.18956177 | 2.42E-04 |
| ADAMTS2 | 0.181119962 | 1.19855895 | 3.47E-04 |
| KIAA0196 | 0.039901858 | 1.04070863 | 1.79E-04 |
| TLCD1 | 0.141979438 | 1.15255295 | 4.26E-06 |
| C1orf95 | 0.162108307 | 1.1759876 | 6.43E-06 |
| PTGER4 | 0.042248674 | 1.04315385 | 4.11E-04 |
| PRKDC | 0.047766939 | 1.04892616 | 1.57E-04 |
| PSMB8 | 0.012162155 | 1.01223641 | 9.76E-04 |
| CPVL | 0.059630966 | 1.06144476 | 3.61E-04 |
| VGF | 0.009815207 | 1.00986353 | 1.16E-04 |
| PIK3C2A | 0.061877445 | 1.06383196 | 6.22E-04 |
| RPS6KA2 | 0.090106486 | 1.0942908 | 1.00E-03 |
| CRABP2 | 0.094361146 | 1.09895656 | 8.01E-05 |
| SQLE | 0.051891946 | 1.05326193 | 2.53E-04 |
| FABP3 | 0.010594016 | 1.01065033 | 3.80E-04 |
| TRPV2 | 0.019591822 | 1.019785 | 5.56E-06 |
| MRC2 | 0.145955266 | 1.15714442 | 2.87E-04 |
| IMPA1 | 0.109422776 | 1.11563391 | 3.54E-04 |
| CCNO | 0.18947809 | 1.20861864 | 4.58E-04 |
| GPR56 | 0.022675565 | 1.02293461 | 1.22E-04 |
| LOXL4 | 0.039128676 | 1.03990429 | 6.23E-04 |
| HTATIP2 | 0.114024035 | 1.12077906 | 1.24E-04 |
| S100A4 | 0.006858938 | 1.00688251 | 1.93E-05 |
| PSMB9 | 0.026735888 | 1.0270965 | 6.10E-04 |
| C1S | 0.054100848 | 1.05559105 | 8.79E-04 |
| LHFPL3 | 0.176362662 | 1.19287059 | 7.93E-05 |
| ANKRD30B | 0.289566673 | 1.3358485 | 3.10E-04 |
| ARHGDIG | 0.324588043 | 1.3834606 | 5.41E-06 |
| CA12 | 0.071066313 | 1.07365242 | 5.55E-07 |
| EEF1A2 | 0.006393623 | 1.00641411 | 1.89E-04 |
| CD48 | 0.200320491 | 1.22179427 | 3.69E-05 |
| POMC | 0.193734782 | 1.21377433 | 4.41E-04 |
| MMP9 | 0.072768232 | 1.07548125 | 1.87E-06 |
| MATK | 0.107561181 | 1.11355899 | 2.38E-05 |
| NR6A1 | -2.560264924 | 0.07728426 | 9.32E-04 |
| SLC25A38 | -0.205369964 | 0.81434598 | 4.01E-05 |
| TFAP2A | -0.036574818 | 0.96408596 | 3.74E-04 |
| ZNF835 | -2.734129058 | 0.06495055 | 5.84E-04 |
| FBXO17 | -0.515604551 | 0.59713948 | 3.39E-04 |
| MANEAL | -1.50186371 | 0.2227147 | 4.77E-04 |
| CAMSAP3 | -0.82189012 | 0.43959997 | 5.20E-04 |
| CTF1 | -0.522217542 | 0.59320363 | 1.31E-04 |
| SLC44A3 | -0.21074634 | 0.8099795 | 5.65E-04 |
| BAP1 | -0.075683299 | 0.92710978 | 1.27E-04 |
| LMCD1 | -1.836271494 | 0.15941068 | 4.51E-04 |
| ALDH1L1 | -0.668650766 | 0.51239946 | 6.96E-04 |
| ACSF2 | -0.478848098 | 0.61949658 | 5.03E-04 |
| LURAP1 | -0.671457465 | 0.51096332 | 1.47E-04 |
| EFS | -0.481604151 | 0.61779157 | 5.97E-04 |
| SEMA3B | -0.504763985 | 0.60364803 | 3.90E-04 |
| RNF208 | -0.541824001 | 0.58168629 | 2.00E-04 |
| MLIP | -0.448837809 | 0.63836963 | 6.81E-04 |
